# Case Report: Endoscopic examination improves the diagnosis of inconspicuous helminth infections in adults in Shanghai

**DOI:** 10.3389/fmed.2025.1541099

**Published:** 2025-05-21

**Authors:** Yang Si, Hongjiao Cai, Brett D. Hambly, Yuli Wang, Yanfang Zhang, Shisan (Bob) Bao

**Affiliations:** ^1^Department of Hematology, Shanghai Ninth People’s Hospital, School of Medicine, Shanghai Jiao Tong University, Shanghai, China; ^2^Division of Laboratory Medicine, Central Hospital of Dalian University of Technology, Dalian, China; ^3^Scientific Research Division, The First People’s Hospital of Baiyin, Baiyin, China; ^4^Third Affiliated Hospital of Gansu University of Chinese Medicine, Lanzhou, China

**Keywords:** hookworm, adult, inconspicuous, endoscopic, metropolitan

## Abstract

With advancements in medical care and improved public health in China, the incidence of hookworm infections has significantly decreased, particularly in first-tier cities. We report a case of severe microcytic hypochromic anaemia caused by hookworm disease. The patient received multiple blood transfusions for unexplained anaemia, with negative faecal smear results. GI endoscopic examination revealed hookworms in the pyloric ring, antrum, and duodenum, which were removed using biopsy forceps. Morphological analysis identified the worms as *Ancylostoma duodenale*. The patient was treated with a single dose of 400 mg albendazole and hematinics. Follow-up haemoglobin testing 3 months later showed an improvement to 126 g/L (115–150 g/L). This case highlights the importance of GI endoscopy diagnostics in identifying a typical presentations of hookworm disease, particularly in first-tier cities. Timely and accurate diagnosis of hookworm infections is essential for preventing long-term health consequences and reducing associated healthcare costs.

## Introduction

Soil-transmitted helminths remain a significant public health concern, particularly in developing countries, with an estimated 1.5 billion people infected at least once in their lifetime worldwide (1). Despite extensive advancements in medical management, neglected tropical diseases persist in China, likely due to inadequate hygiene practices, particularly in rural areas (2).

Historically, soil-transmitted helminth infections were widespread in China during the 1950s–1960s but have been successfully controlled through significant governmental efforts (2). Consequently, hookworm infections have become extremely rare (3), and many medical practitioners are now unfamiliar with the disease.

The primary species responsible for hookworm infections include *Necator americanus*, *Ancylostoma duodenale*, and *Ancylostoma ceylanicum*, which are commonly found in tropical and subtropical regions (4). While hookworm infections were historically prevalent in China, improvements in healthcare and sanitation have significantly reduced their occurrence, particularly in urban areas. As of 2019, the national infection rate was 0.85%, with a weighted prevalence of 0.66%. However, some provinces, such as Sichuan (4.75%), Chongqing (2.54%), and Hainan (2.44%), still report relatively high infection rates (3).

Factors influencing infection rates include age, sex, sanitation, and lifestyle (5). The severity of hookworm infection depends on worm burden as well as the host’s nutritional status, immunity, and overall health (6). Clinical manifestations are often non-specific, leading to misdiagnoses and delays in treatment.

Due to the rarity of hookworm infection in modern clinical practice, particularly in first-tier cities, most physicians are unfamiliar with its presentation. In the case described here, physicians at the Ninth People’s Hospital opted for an endoscopic examination after other diagnostic methods failed to identify the cause. Although gastrointestinal endoscopy is not a standard routine diagnostic tool for hookworm infection, this case underscores the need for increased awareness among medical practitioners. By presenting this case, we aim to highlight the importance of considering hookworm infection in patients with unexplained iron deficiency anaemia, particularly in individuals from endemic regions.

## Case report

A 60-year-old female presented with severe iron deficiency anaemia of unknown origin, reporting fatigue, malaise, and loss of appetite for 6 months. She had recently been living in rural Sichuan, where she had received multiple red blood cell transfusions at a local hospital for persistent anaemia before being referred to our hospital in Shanghai. Physical examination revealed extreme pallor without other notable findings. The patient initially received medical care at Guang’an District People’s Hospital, Guang’an City, Sichuan Province. However, due to uncertainty regarding the underlying condition, she was referred to the Ninth People’s Hospital, Shanghai Jiao Tong University School of Medicine, for further evaluation.

Initial laboratory tests revealed the following results: red blood cell count, 2.84 × 10^12^/L (normal: 3.8–5.1 × 10^12^/L); haemoglobin, 38 g/L (normal: 115–150 g/L); haematocrit, 20.7% (normal: 35–45%); mean corpuscular volume (MCV), 59.6 fL (normal: 82.9–98 fL); mean corpuscular haemoglobin (MCH), 15.1 pg (normal: 27–34 pg); and mean corpuscular haemoglobin concentration (MCHC), 253 g/L (normal: 316–354 g/L). Eosinophilia (27.1%; 1.49 × 10^9^/L) (normal: 0.4–8%; 0.02–0.52 × 10^9^/L) suggested a parasitic or allergic process (Supplementary Table 1).

Liver and renal function tests, urinalysis, and stool analysis revealed no abnormalities, and no eggs or worms were observed in the faeces under microscopy. The faecal specimens were examined three times under a microscope; however, the faecal occult blood test was positive. Additionally, the patient was not immunocompromised and had not received any other medications. Interestingly, no eggs were detected in the faeces, which puzzled the clinicians and prompted an endoscopy. A possible explanation is that both the clinicians and laboratory staff were inexperienced in diagnosing hookworm in a hospital from a first-tier city, despite it being a teaching hospital.

**Figure 1:**
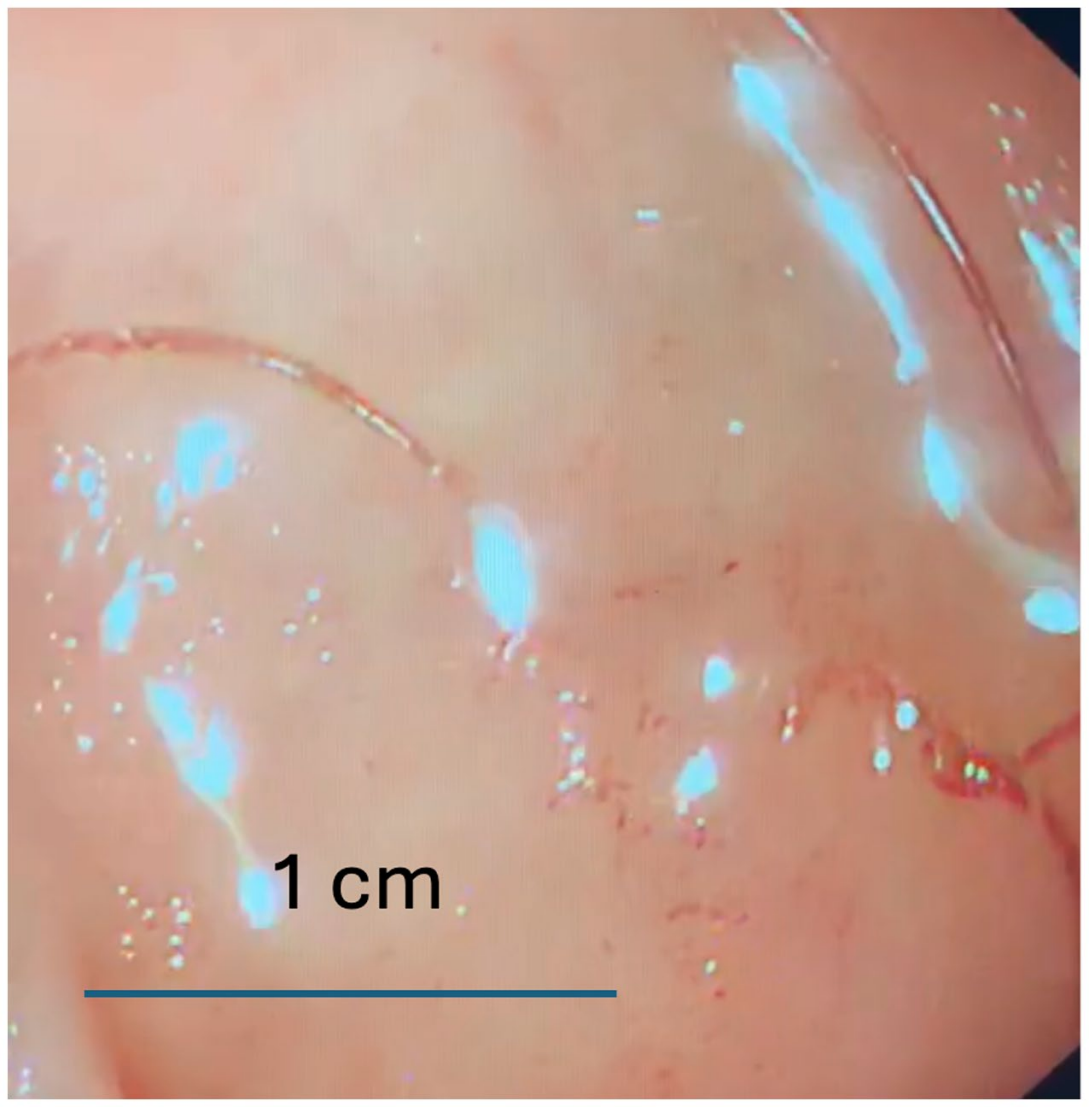
Endoscopic detection of hookworm in the pyloric ring from the patient.

**Figure 2:**
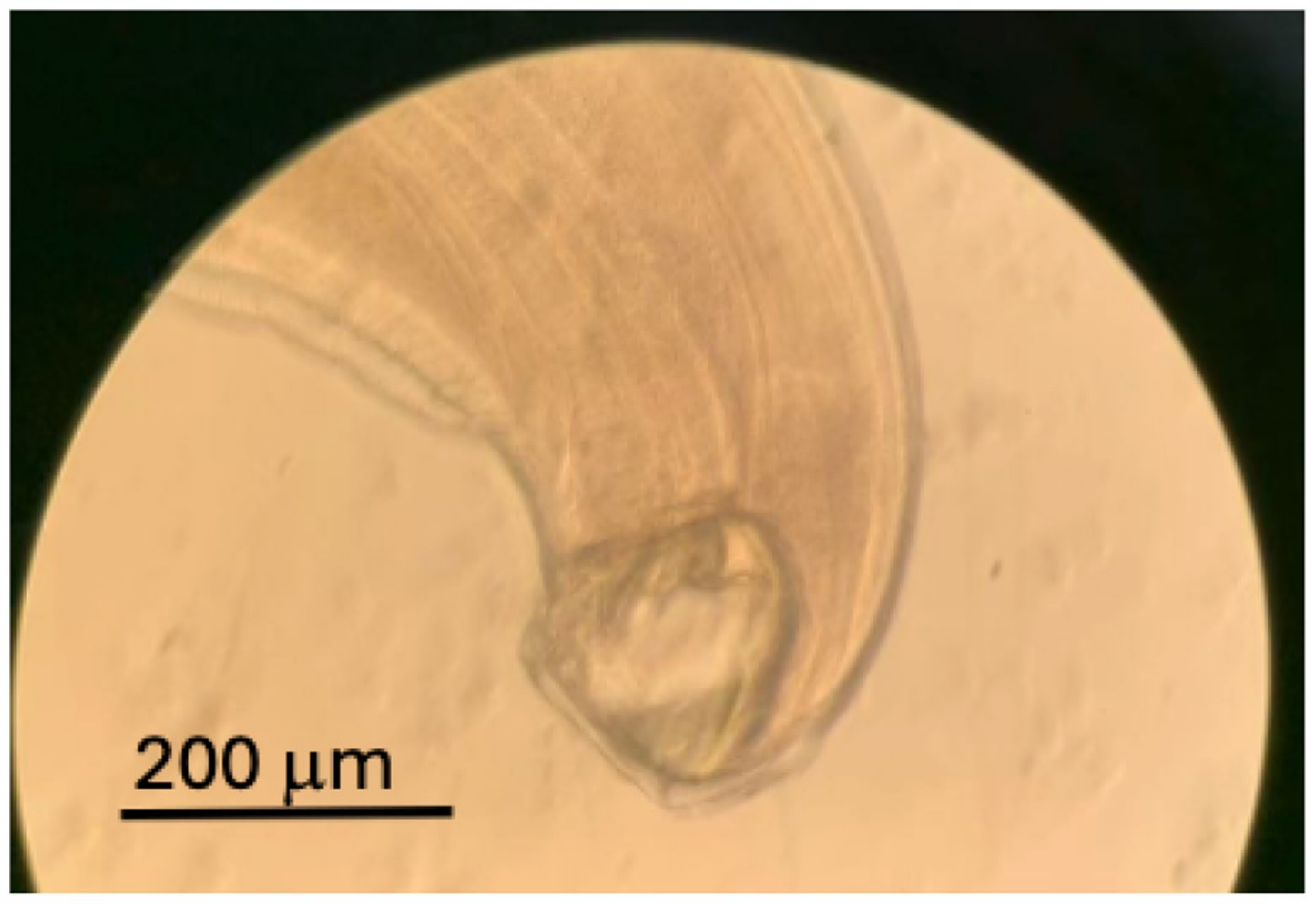
Typical hookworm infection was confirmed through microscopic examination of the worm captured via endoscopy.

To investigate further, gastroscopy and colonoscopy were performed. While colonoscopy yielded no significant findings, gastroscopy identified living worms in the pyloric ring, antrum, and duodenum, along with areas of active bleeding (Figure 1). The worms were removed using biopsy forceps and were morphologically identified as *Ancylostoma duodenale*, a finding confirmed by microscopic examination (Figure 2).

The patient was treated with a single 400 mg dose of albendazole and hematinics. Her haemoglobin level improved to 85 g/L at discharge and normalized to 126 g/L after 3 months.

## Discussion

Hookworm is among the most common soil-transmitted helminths, residing in the host’s small intestine and causing iron deficiency anaemia (IDA) through chronic blood loss (7). Its life cycle begins when third-stage larvae (L3) penetrate human skin from contaminated soil, enter the bloodstream, and travel to the lungs. The larvae subsequently migrate to the pharynx, are swallowed, and mature into adult worms in the small intestine, where they attach to the mucosa, feed on blood, and reproduce, releasing eggs that are expelled through faeces (8).

Timely diagnosis and treatment of hookworm infection are crucial for preventing severe complications. Diagnosis is typically made by detecting characteristic eggs in stool samples via microscopy. However, this method requires technical expertise and is prone to false negatives (9), particularly in low-prevalence areas such as this one. More recently, emerging technologies such as automated digital stool analysers have improved diagnostic accuracy but are not widely available, perhaps due to cost (10). Additionally, gastrointestinal endoscopy is increasingly used as a complementary diagnostic tool, particularly in atypical cases or when stool tests yield negative results (11, 12). However, capsule endoscopy appears to be a more versatile option with fewer potential complications or unintended outcomes (7).

Molecular diagnostics, such as mitochondrial genome sequencing (13), have also proven valuable in accurately identifying hookworm species, which is critical for selecting appropriate anthelmintic treatment and advancing epidemiological research (14, 15). However, cost-effectiveness should be considered, as spontaneous tube sedimentation (STS) diagnostic techniques have demonstrated both accuracy and affordability, especially in the developing countries (16). Accurate identification is particularly important when specimens are incomplete, as different species exhibit variable drug sensitivities.

In this case, substantially delayed diagnosis was attributed to the patient’s relocation from rural Sichuan to Shanghai, which confused doctors in an area where awareness of hookworm infection is limited. This case highlights the importance of thorough diagnostic workups and the need for increased vigilance among urban healthcare providers.

Furthermore, for public health and epidemic control, the WHO recommends administering anthelmintic medications to children (17) and implementing combined treatment strategies for schistosomiasis and soil-transmitted helminthiasis in high-prevalence regions, particularly in Africa (18). Although significant efforts have been made to minimise or control helminthic transmission and infection through large-scale deworming and rebuilding sanitation systems, challenges persist, particularly in the poorest regions of central China, where economic hardship is exacerbated by mountainous and desert terrain (19). Given this case, it may be advisable to extend such preventative measures to adults in rural Chinese regions with a higher parasite prevalence, as recommended by the WHO (18). Despite stool examination is easy and reliable if it is handled by the experienced staff, but our case demonstrated further training is necessary for the medical practitioners, and prepared for any unprepared. More recently, PCR testing has been applied in diagnosis of hookworm infection, improving substantially in sensitivity and specificity (20). Such approach probably will be very useful in hospitals from first-tier cities.

## Conclusion

Timely and accurate diagnosis of worm infection is essential for minimizing long-term health consequences and reducing associated healthcare costs. This case highlights the utility of gastrointestinal endoscopy in identifying a typical presentations of hookworm disease. Preventative measures, including deworming programmes for children and potentially adults, remain vital in controlling hookworm infections and mitigating their public health impact.

## Data Availability

The original contributions presented in the study are included in the article/Supplementary material, further inquiries can be directed to the corresponding authors.
